# Quels agents incriminés dans les mycoses du pied ? Enquête auprès des diabétiques consultant au CHU Mohammed VI de Marrakech

**DOI:** 10.11604/pamj.2014.17.228.3131

**Published:** 2014-03-26

**Authors:** Hakima Chegour, Nawal El Ansari, Ghizlane El Mghari, Abdelali Tali, Laila Zoughaghi, Majda Sebbani, Mohamed Amine

**Affiliations:** 1 Service d’‘Endocrinologie Diabétologie et maladies Métaboliques, Maroc, Laboratoire de recherche de Pneumo-Cardio-Immunopathologie et Métabolisme, CHU Mohammed VI Marrakech, Faculté de Médecine et de Pharmacie de Marrakech, Université CaddiAyad, Marrakech, Maroc; 2 laboratoire de mycologie parasitologie, CHU Mohammed VI Marrakech, Maroc; 3 laboratoire d’épidémiologie, laboratoire de recherche de pneumo-cardio-immunopathologie et métabolisme, Faculté de Médecine et de Pharmacie de Marrakech, UCAM

**Keywords:** Diabète, examen mycologique, mycose, onycomycose, pied diabétique, diabetes, mycological examination, mycosis, onychomycosis, diabetes foot

## Abstract

Les infections mycosiques du pied constituent un motif fréquent de consultation chez les diabétiques, le diabète constituant à la fois un facteur favorisant et aggravant les lésions cutanéomuqueuses. L'objectif de ce travail était d'identifier la flore mycologique locale responsable des lésions du pied chez le diabétique et déterminer les facteurs favorisant la survenue de mycoses. Il s'agissait d'une étude transversale intéressant des diabétiques suivis en consultation; un prélèvement mycologique, avec examen direct et culture, a été réalisé devant toute suspicion clinique de lésion mycosique. Quatre-vingt-deux patients ont été inclus. L'hémoglobine glycosylée moyenne a été de9,2% ± 2,23. Un intertrigo inter orteil a été noté dans 90,2% des cas; l'examen mycologique était positif dans 64,8% des cas, avec 18 cas de *Trichophyton rubrum* et 11 cas de *Candida albicans*. Une atteinte unguéale a été suspectée chez 65,9% patients; la culture a mis en évidence un Trichosporon pathogène chez sept patients, un *Candida albicans*dans six cas, un *Trichophyton rubrum* dans quatre cas, avec trois cas de *Trichophyton mentagrophytes* et deux cas de *Scytalidium dimidiatum*. L’étude analytique, après confirmation mycologique, en fonction des principales caractéristiques des patients a montré que l'atteinte mycosique du pied est significativement corrélée au déséquilibre glycémique. Ce travail a montré la prédominance du *Trichophyton rubrum* dans les lésions d'intertrigo inter orteil et du Trichosporon dans les onychomycoses, avec une prédominance globale plus globale plus élevée du TR.

## Introduction

Les infections mycosiques du pied constituent un motif fréquent de consultation, leur prévalence semble plus élevée chez les diabétiques par rapport à la population générale [1, 2]. Les agents fongiques trouvent sur ce terrain les conditions idéales à leur développement et exposent aux risques de surinfection bactérienne avec risque d’évolution vers la gangrène et l'amputation. La flore mycologique locale responsable de ces lésions est peu connue, l’étude du profil mycologique s'avère d'un intérêt majeur surtout en terme de prise en charge thérapeutique. Ce travail permettra de confirmer le diagnostic de mycoses par les prélèvements mycologiques et d'avoir un profil mycologique précis, pouvant guider la prescription médicamenteuse. Objectifs et contexte: L'objectif de la présente étude est d'identifier les agents fongiques incriminés dans les lésions mycosiques du pied chez le diabétique et de déterminer les facteurs favorisant la survenue de mycoses. À notre connaissance et jusqu’à la réalisation de ce travail aucune étude similaire n'a été mené localement dans ce sens.

## Méthodes

Il s'agit d'une étude transversale à visée descriptive. La population d’étude a été formée par un échantillon représentatif de patients diabétiques vus en consultation de Diabétologie au CHU Mohammed VI de Marrakech entre Août 2010 et Janvier 2011, présentant des lésions cutanées des pieds évocatrices d'atteinte mycosique. Ils ont été exclu de ce travail, les patients présentant des signes d'infection bactérienne au niveau du pied (écoulement suintant, présence de pus), et ceux ayant reçu un traitement antifongique par voie locale (crème antifongique ou kératolytique) depuis moins de 15j ou moins de trois mois après application d'une solution filmogène ou prise d'antifongique par voie orale. Les données ont été collectées à travers une fiche d'exploitation, par un médecin à l’échelle du service d'Endocrinologie Diabétologie et Maladies métaboliques.

Au cours de ce travail les définitions opérationnelles suivantes ont été adoptées: Atteinte mycosique des espaces inter orteils; Atteinte mycosique des ongles; Atteinte mycosique de la plante; L’équilibre glycémique a été jugé par l'hémoglobine glycosylée (HbA1c): Ils ont été considéré équilibré les patients avec une HbA1c inférieure à 7%, moyennement équilibré quand l'HbA1c est entre 7 et 8% et déséquilibré quand celle-ci est supérieur à 8%. Un examen clinique des pieds a été systématiquement effectué, à la recherche d'anomalies cliniques évocatrices de mycose des ongles, des plis inter orteils ou des plantes. Devant chaque suspicion clinique, un prélèvement mycologique a été effectué au niveau des trois sites (ongles, espaces inter orteils(EIO) et plante), avec recherche de mycoses associées. L'examen mycologique comportait un examen direct et une culture pratiquée au laboratoire de mycologie du CHU Med VI de Marrakech. La mise en culture des micromycètes a été réalisée par un ensemencement sur milieux de Sabouraud; Sabouraud-chloramphénicol pour inhiber les bactéries et Sabouraud-chloramphénicol et cycloheximide pour inhiber les champignons saprophytes. L'examen mycologique est considéré positif quand l'examen direct et/ou la culture sont positifs. Les données recueillies ont été saisies et analysées sur le logiciel Epi info. Nous avons eu recours au test de Khi2 pour comparer les pourcentages et au test de Student pour comparer les moyennes. Nous avons pris comme seuil de significativité p = 0,05.

## Résultats

**Figure 1:**
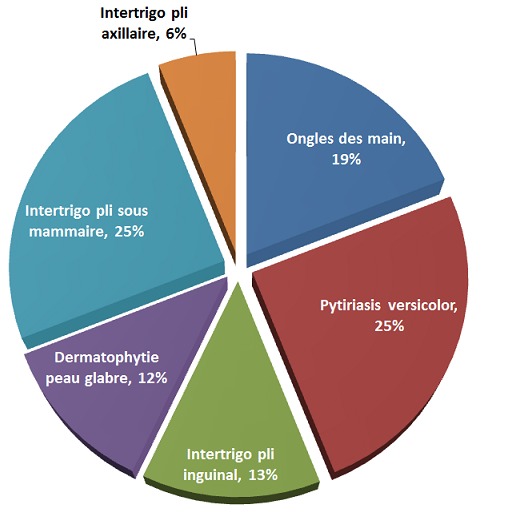
Autres lésions mycosiques associées

Durant la période étudiée, 82 patients vus en consultation présentaient des lésions du pied suspectes de mycose, le Tableau 1 résume les caractéristiques des patients. La moyenne d’âge des patients a été de 56 ans ± 11,1ans, le sexe ratio (F/H) a été de 1,5. Le diabète a été de type 2 dans 93% des cas, l'ancienneté moyenne du diabète a été de 8 ans ±6,58. L'HbA1c n'a étéréalisé que par 67 patients, l'HbA1c moyenne était de: 9,2% ± 2,23, le diabète était moyennement équilibré dans 17,9% des cas, mal équilibré dans 64,1% des cas (43 patients) et bien équilibré chez 12 patients. L'indice de masse corporelle (IMC) moyen a été de 26,6 ± 4,8 kg/m2. Les résultats de l'examen mycologique (examen direct et culture) sont représentés dans les Tableau 2 et Tableau 3. Des lésions mycosiques associées aux lésions du pied ont été retrouvées dans 29 cas (35,3%), dont 3 cas d'onychomycoses des mains, 10 cas de candidose buccale, 5 cas de candidose vaginale, 11 cas de lésions mycosiques des intertrigos et de la peau glabre et 3 cas de Pityriasis versicolor (Figure 1).

**Tableau 1:** Caractéristiques de la population étudiée

	Nombre de patients	Pourcentages%
**Type diabète**		
Type1	6	7
Type2	76	93
Hypertension artérielle	35	43
Dyslipidémie	26 (n = 44)	59
Antécédents podologiques	36	43,3
IIO	31	86
Mal perforant plantaire	1	3
Amputation	4	11
Obésité	25	30,5
Surpoids	23	28
Rétinopathie	25	50 (n = 50)
Néphropathie	18	33,3 (n = 54)
Cardiopathie ischémique	12	18,2 (n = 66)
Artérite des membres inférieurs	3	3,6
Neuropathie périphérique	51	62,2(n=82)

**Tableau 2:** Siège des lésions et résultats de l'examen direct

Siège de la lésion	Nombre de patients	Pourcentage%	Examen direct			
			FM	LB	FM + LB	Négatif
Espace inter orteil	74	90,2	35	13		34
Atteinte unguéale	54	65,9	18	13		51
Atteinte plantaire	30	36,6	3	2	2	77

**LB**=levures bourgeonnantes **FM**=filaments mycéliens

**Tableau 3:** Résultats de la culture

Siège	Négative		Positive										
	Nombre	%	Nombre	%	TR	TM	CA	CNA	TP	AF	SD	Ssp	OC
I IO	34	41,5	48	58,5	18	4	11	4	6	1	1	1	2
Atteinte unguéale	56	68,3	26	31,7	4	3	6	1	7	1	2	1	1
Atteinte plantaire	71	86,6	11	13,4	2	2	1	1	3	1	0	1	0

**TR** : Trichophyton rubrum ; **AF** : Aspergillusflavus ; TM : Trichophyton mentagrophytes ; **SD** : Scytalidiumdimidiatum ; **CA** :Candida albicans ; **Ssp** :scopularissp ;**CNA** :Candida non albicans ; **OC** :onychocolacanadensis ;**TP** :Trichosporon ; **EIO** : espace inter orteil

L’étude analytique, après confirmation mycologique, en fonction des principales caractéristiques des patients a montré que l'atteinte mycosique du pied est significativement corrélée audéséquilibre glycémique. Les mycoses des piedsont été observées chez 32 hommes (39%) et 50 femmes (61%), la différence était non significative. Il n'y avait pas d'association significative avec le type du diabète, les anomalies du bilan lipidique ou encore avec l'existence de complications dégénératives, notamment une neuropathie périphérique (Tableau 4). En considérant les résultats de l'examen direct et la culture au niveau des trois localisations, l'examen direct semble avoir une faible sensibilité (27,3%) dans le diagnostic positif des mycoses plantaires (Tableau 5).

**Tableau 4:** Facteurs associés aux lésions mycosiques des pieds

	Pourcentage des patients%	P
Diabète mal équilibré	64,1(n = 67)	0,004
Dyslipidémie	59	NS
Surcharge pondérale	28	NS
Obésité	30,5	NS
Rétinopathie	50(n = 50)	NS
Neuropathie périphérique	62,2 (n = 82)	NS
Cardiopathie ischémique	18,2 (n = 66)	NS
Artérite des membres inférieurs	3,6	NS
Age > 50ans	68	NS
Ancienneté du diabète >10ans	34	NS

**NS**=non significatif

**Tableau 5:** Corrélation des résultats de l'examen direct et de la culture

	Sensibilité%	Spécificité%
Intertrigo inter-orteil	77,1	69,6
Onychomycose	80,8	82,1
Atteinte plantaire	27,3	97,2

## Discussion

Le pied diabétique est un problème majeur de santé publique comme l′attestent les données épidémiologiques. Le taux d′amputations de membres inférieurs reste très élevé même dans les pays de haut niveau socioéconomique [3]. L'infection, élément de gravité, est la 3èmecomposante du trépied physiopathologique du pied diabétique après la neuropathie et l'artériopathie. Elle est exceptionnellement à l'origine directe d'une plaie. Seule une mycose interdigitale peut devenir creusante par surinfection bactérienne [4, 5]. L'atteinte mycosique est fréquente chez le diabétique, l'onychomycose est une affection dont la prévalence est comprise entre 8 et 10% de la population générale. Chez le diabétique, la prévalence est plus élevée, elle varie entre 18,7% [6] et 22% [7]. Le diabète par ses conséquences sur la microcirculation peut faciliter la survenue d′une onychomycose [8]. Les déformations de la tablette unguéale qu'elles provoquent, pourraient, chez les diabétiques, être un facteur de risque de mal perforant plantaire, et pourraient, indirectement, favoriser la récidive d’érysipèles, par recontamination des intertrigos interdigito plantaires [8].

Dans ce travail, parmi les 82 patients suspects d'atteinte mycosique du pied, la lésion unguéale a été confirmée par l'examen mycologique (l'examen direct et/ou la culture) chez 32 patients parmi 54 (59,2%), l'intertrigo inter-orteil chez 51 patients parmi 74 suspecté cliniquement (68,9%), alors que l'origine mycosique de l'atteinte plantaire n'a été retenue que chez 11 patients parmi 30 (36,6%). Le siège de prédilection a été les plis inter-orteils, suivi par les ongles et les plantes des pieds. Le Trichophyton rubruma été le principal agent en cause des lésions mycosiques (28,23%), Il a été retrouvé sur 24 cultures parmi les 85 cultures positives. Dans cette étude, les mycoses ont été significativement plus fréquentes chez les diabétiques mal équilibrés. D′après une étude réalisée par Buxon à Edinburg, en Grande-Bretagne [9], la prévalence des onyxis des pieds à dermatophytes serait de 12% chez le patient diabétique alors qu′elle est à 11% dans la population générale, une différence négligeable. En revanche, une autre étude, réalisée conjointement à Londres, en Ontario au Canada et à Boston aux USA [10] portant sur 550 patients diabétiques, révèle un taux d′onychomycose 2,7 fois plus élevé que dans la population contrôle du même âge, avec une fréquence 3 fois plus élevée chez l′homme par rapport à la femme. Le risque relatif d'onyxis des pieds chez le diabétique a été évalué dans plusieurs études, et serait compris entre 1,5 et 2,14 et celui des mycoses des plis inter-orteils entre 1,48 et 2,1 [11, 12]. Si le diabète peut être un facteur favorisant en raison des troubles trophiques et du déficit circulatoire qu′il engendre, il représente visiblement un facteur aggravant (risque de surinfection, érysipèle) qu′il convient de prendre en compte et de traiter efficacement [3].

La revue de la littérature, montre une discordance entre l'examen clinique et l'examen mycologique, résultat retrouvé par la plupart des auteurs. Lugo et al, en étudiant 100 patients diabétiques trouvaient une prévalence de 73% des mycoses superficielles à l'examen clinique contre 34% à l'examen mycologique [13]. Une étude tunisienne sur 150 patients a objectivé une prévalence clinique des mycoses du pied de 57,3% versus 54% à l'examen mycologique [14]. Dans la ci présente étude, la discordance entre l'examen clinique et l'examen mycologique paraît évidente mais la prévalence clinique des lésions mycosiques dans la population étudiée ne peut être précisée vu que l’étude s'est intéressée seulement aux patients avec signes cliniques d'atteinte mycosique. Le siège de prédilection était les plis inter-orteils, suivi par les ongles et les plantes des pieds; ce même profil est retrouvée dans le travail d'El Fékih et al, avec une fréquence des mycoses des plis inter-orteils de 72,8%, suivie par les onychomycoses (56,7%) et l'atteinte plantaire (19,7%). Le déséquilibre du diabète est en effet souvent associé aux infections fongiques du pied [10, 15], il est significativement corrélé aux onychomycoses [16]. Dans cette étude, les mycoses ont été significativement plus fréquentes chez les patients présentant un diabète mal équilibré. Le déséquilibre du diabète, peut constituer un facteur associé à la survenue de mycose des pieds. Dans ce travail, seulement 17,9% des patients avaient une HbA1c ≤ 7% avec une majorité de patients déséquilibrés (HbA1c moyenne à 9,2 ± 2,23%), ce qui pourrait être expliqué par le fait que la population étudiée est représentée par des patients adressés aux CHU pour déséquilibre glycémique d'un diabète multi compliqué. La prédisposition particulière des patients présentant un diabète ancien à développer des mycoses des pieds est controversé [10, 15, 17] et n'a pas été retrouvée dans cette série. La neuropathie périphérique, en raison des troubles trophiques qu'elle peut engendrer est considérée par plusieurs auteurs comme un facteur favorisant l'infection mycosique chez le diabétique [12, 18, 19], ceci n'a pas été trouvé dans ce travail. Le rôle de l'artérite des membres inférieurs [10, 12], du sexe masculin [10, 17, 20] et de l'obésité est différemment apprécié dans la littérature [10, 12, 21]; dans ce travail, aucune corrélation significative n'a été retrouvée concernant ces paramètres. L'hypertriglycéridémie est souvent associée à une l'hypo-HDL-cholestérolémie, à une hyperglycémie, à un hyperinsulinisme et à une obésité. De ce fait, elle a été supposée comme facteur favorisant les mycoses des pieds; cette association n'a pas été notée dans ce travail ni d'autres études similaires [10, 12, 22]. Dans une étude récente, la prévalence de l'onychomycose était significativement corrélée à l'existence d'une rétinopathie [23]. Enfin, aucune relation n'a été mise en évidence entre l'hypertension artérielle, l'insuffisance coronarienne et la néphropathie diabétique et la mycose des pieds [15, 22]. Les dermatophytes, les levures du genre Candida et les moisissures se partagent les étiologies des lésions mycosiques du pied [24, 25]. Les autres levures (Torulopsis, Trichosporon) sont exceptionnellement en cause. Les dermatophytes prédominent largement (plus de 90%). Le Trichophyton rubrum(TR) constitue plus de 70% des dermatophytes isolés; Trichophyton mentagrophytes, variété interdigitale (25%), plus rarement Epidermophytonfloccosum, Microsporumsp.

Dans cette étude, le Trichophyton rubrum était le principal agent en cause des lésions mycosiques (28,2%), Il a été retrouvé sur 24 cultures parmi les 85 cultures positives, cela a d'ailleurs été décrit dans d'autres séries [14, 15, 26]. Cet agent prédomine dans les lésions d'intertrigos inter-orteils avec une fréquence de 24,3%. La fréquence des infections à Trichophyton mentagrophytes (TM)est variable, elle serait pour certains auteurs identique chez le diabétique et le non diabétique [27]; pour d'autres 45% des onychomycoses chez le diabétique seraient secondaires à TM [10], dans cette série, la fréquence des mycoses à TM était seulement de 10,6%. Le Candida Albicans est associé à 20% des mycoses dans ce travail contre 10,5% dans la série d'El Fékih [14]. Les moisissures sont rarement responsables (moins de 10% des onychomycoses); elles sont saprophytes du milieu extérieur et parasitent volontiers un ongle déjà pathologique ou infecté par un dermatophyte et dont le caractère pathogène doit être solidement argumenté par un examen mycologique et/ou histologique rigoureux; il s′agit principalement de Scopulariopsis (surtout brevicaulis), Aspergillus (Aspergillus versicolorsurtout), Fusarium (oxysporum surtout), Acremonium et aussi de Scytalidiumdimidiatum, Scytalidiumhyalinum, au comportement proche des dermatophytes. Les Scytalidium sont plus fréquemment isolés chez des patients venant des régions tropicales (Antilles par exemple, Afrique, Inde, Pakistan,…). [1, 28].

Par ailleurs, il a été rapporté des infections à champignons saprophytes seuls ou associés à des dermatophytes [1, 19]; dans le travail ci présent, les levures du genre Trichosporon, agent saprophyte habituellement non pathogène, ont été retenu comme agent fongique responsable de 18,8% des atteintes unguéales et plantaires, le caractère pathogène était évoqué devant l'isolement dans deux ou trois sites prélevés. Le genre Trichosporon regroupe des levures vivant en commensales sur le revêtement cutané, qui se caractérisent par un mycélium et un pseudo-mycélium abondant. LeTrichosporonasahii, espèce la plus isolée en pathologiehumaine, est responsable de septicémies ou d'infections profondes. Trichosporonasteroïdeset T. cutaneum ont été impliqués dans des lésions d'onyxis, d'intertrigos. Il s'agit de levures opportunistes, pour affirmer le caractère pathogène d'un isolat, l'interprétation sera avantageusement facilitée par une confrontation clinico-biologique après répétition des prélèvements mycologiques [29]. Des lésions mycosiques incriminant des agents rarement pathogène et rarement rapportés dans la littérature ont par ailleurs été notées [30, 31], il s'agit de moisissures: ScopularisSp, ScytalidiumDimidiatum et de nouveau pseudodermatophytesemergent: OnychocolaCanadensis qui parasite plus particulièrement les ongles des orteils de la femme âgée ayant des troubles trophiques et vasculaires. Une trentaine de cas d'Onychocola, dont on ne connaît pas le biotope naturel, sont actuellement recensés dans la littérature spécialisée [32]. Compte tenu des résultats de ce travail et des risques graves que peut engendrer une atteinte mycosique négligée chez un diabétique, un traitement par la terbinafine visant le TR devra être prescrit le plutôt possible devant des lésions d'intertrigo interorteil après réalisation d'un prélèvement mycologique qui reste indispensable dans les formes disséminées ou récidivantes. Concernant l'atteinte unguéale et plantaire, l'examen mycologique devrait être systématique, avant de démarrer un traitement souvent long et couteux, vu que l'atteinte des ongles est souvent multifocale et que le profil mycologique retrouvé dans ce travail est particulier par la prédominance des lésions à Trichosporonspp.

Le traitement des onychomycoses reste difficile et nécessite un long suivi; les nouvelles molécules terbinafine et itraconazole, les solutions filmogènes (amorolfine, ciclopirox) paraissaient prometteuses. La terbinafine et l′amorolfine peuvent être prescrites en première intention dans les infections dermatophytiques, l'itraconazole est un excellent choix quand il s'agit d'une infection à levures opportunistes [33], alors que lefluconazolereste la troisième alternative possible.

## Conclusion

Ce travail a montré la prédominanceduTrichophyton rubrum dans les lésions d'IIO et duTrichosporon dans les onychomycoses, avec une prédominance globale plus élevée du TR, ces résultats rejoignent en partie les données de la littérature, la majorité des séries rapportent la prédominance de l'atteinte dermatophytique même pour les onychomycoses, contradictoirement aux résultats de la présente étude qui a objectivé un taux plus important d'onychomycoses à Trichosporonpathogène. L'examen mycologique devrait complémentaire de l'examen clinique puisqu'il ne permet pas à lui seul d’établir le diagnostic de mycose et d'en déterminer la flore responsable. Ce travail reste limité par le faible nombre de patients et les biais de recrutement, des études complémentaires chez les diabétiques consultants en dehors des structures hospitalières s'avèrent intéressantes et d'un grand apport pour conforter les résultats de ce travail.

## References

[1] Abu-Elteen KH (1999). Incidence and distribution of candida species isolated from human skin in Jordan. Mycoses..

[2] Bousquet Rouaud R (1994). Mycose et diabète. Glucorama..

[3] Ha Van G, Hartemann A, Haddad J, Bensimon Y, Ponseau W, Baillot J (2011). Pied diabétique. Podologie.

[4] Malgrange D (2008). Physiopathologie du pied diabétique. La revue de médecine interne.

[5] Lipsky BA (2008). Infectious problems of the foot in diabetic patients. Levin and O'Neals The Diabetic Foot.

[6] Al-Mutairi N, Eassa BI, Al-Rqobah DA (2010). Clinical and mycologic characteristics of onychomycosis in diabetic patients. Acta DermatovenerolCroat..

[7] Saunte Ditte Marie L, Jane B Holgersen, Merete Hædersdal, Gitte Strauss (2006). Prevalence of toe nail onychomycosis in diabetic patients. Acta Derm Venereol.

[8] Lugo-Somolinos A, Sánchez JL (1992). Prevalence of dermatophytosis in patients with diabetes. J Am Acad Dermatol..

[9] Buxton PK, Milne LJR, Prescott RJ, Proobfoot MC, Stuart FM (1996). The prevalence of dermatophyte infection in well-controlled diabetics and the response to trichophyton antigen. Br J Dermatol..

[10] Gupta AK, Konnikov N, Mac-Donald P, Rich P, Rodger NW (1998). Prevalence and epidemiology of toenail onychomycosis in diabetic subjects: a multicentre survey. Br J Dermatol..

[11] Roseeuw D, Katsambas A, Burzykowski T, Molenberg G, Marynissen G (1999). The risk of fungal foot infection in diabetic patients. J EurAcadDermatolVenereol..

[12] Gupta AK, Humk S (2000). The prevalence and management of onychomycosis in diabetic patients. Eur J Dermatol..

[13] Lugo-Somolinos A, Jorge LS, San J, Puerto R (1992). Prevalence of dermatophytosis in patients with diabetes. JAmAcadDermatol..

[14] El Fékih N, Fazaa B, Zouari B, Sfia M, Hajlaoui K, Gaigi S, Kamoun MR (2009). Les mycoses du pied chez le diabétique: étude prospective de 150 patients. Journal de Mycologie Médicale.

[15] Jemli B, Essaies O, Derbel S, Gargouri S, Zidi B, Amor A (2003). Mycose du pied et diabète. Feuillets de Biologie..

[16] Takehara K, Tsunemi Oe M, Nagase Y, Ohashi T, Iizaka Y, Ueki S, Tsukamoto K (2011). Factors associated with presence and severity of toenail onychomycosis in patients with diabetes: a cross-sectional study. Int J Nurs Stud..

[17] Wanke NCF, Ave BR, Wanke B, Monteiro PCF, Vastro ACL, Perez MA (1992). Fungal infections in the feet of diabetic patients. J Mycol Med..

[18] Rich P, Oregon P (1996). Special patient populations: onychomychosis in the diabetic patient. J Am AcadDermatol..

[19] Vazquez DS, Jack DS (1995). Fungal infections in diabetes. Infect Dis Clin North Am..

[20] Khorchani H, Haouet H, Amri M, Zanned I, Babba H, Azaiz R (1996). Profil épidémiologique et clinique des mycoses superficielles dans la région de Monastir (Tunisie): Étude rétrospective (1991-1994) à propos de 3578 cas. ArchInst Pasteur Tunis..

[21] Feuillade de Chauvin M (2000). Mycoses métropolitaines. Encycl Med Chir Dermatologie.

[22] Garcia HL, Yergres NR, Blanco MP, Yegres F (2005). Superficial mycose: comparative study between type 2 diabetic and nondiabetic control group. Invest Clin..

[23] Gulcan A, Gulcan E, Oksuz S, Sahin I, Kaya D (2011). Prevalence of toenail onychomycosis in patients with type 2 diabetes mellitus and evaluation of risk factors. J Am Podiatr Med Assoc..

[24] Nenoff P, Ginter-Hanselmayer G, Tietz HJ (2012). Fungal nail infections--an update: Part 1--Prevalence, epidemiology, predisposing conditions, and differential diagnosis. Hautarzt..

[25] Singal A, Khanna D (2011). Onychomycosis: Diagnosis and management. Indian J Dermatol Venereol Leprol..

[26] Monteiro F, Ave RB, Castro L, Perez A, Sacks K, Wanke B (1992). Fungal infection in the diabetic feet of diabetic patients. J Dermatol Med..

[27] Benamor S, Senet P, Chosidow O (2002). Manifestations cutanéomuqueuses du diabète. Encycl Med ChirDermatologie.

[28] Baran R, Bazex J, Baixench MT, Dordain BML (1996). Onychomycoses à Fusarium. Ann Dermatol Venereol..

[29] Chabasse D, Avenel-Audran M, Bouchara JP, Cimon B (1997). Deux nouveaux cas français d'onyxis à Onychocolacanadensis: Etude mycologique. Journal de mycologie médicale..

[30] Chabasse D, Baran R, Feuilhade de Chauvin M (2000). Les onychomycoses I - Épidémiologie-Étiologie. Journal de Mycologie Médicale. décembre.

[31] Chabasse D, Pihet M, Bouchara J-P (2009). Émergence de nouveaux champignons pathogènes en médecine: revue générale. Revue Francophone Des Laboratoires.

[32] Contet-Audonneau N, Schmutz JL, Basile AM, de Bièvre C (1997). A new agent of onychomycosis in the elderly: Onychocolacanadensis. Eur J Dermatol..

[33] Mayser P, Freund V, Budihardja D (2009). Toenail onychomycosis in diabetic patients: issues and management. Am J Clin Dermatol..

